# Musculoskeletal Injury and Illness Patterns in British Eventing Horses: A Descriptive Study

**DOI:** 10.3390/ani14182667

**Published:** 2024-09-13

**Authors:** Carolyne A. Tranquille, Kate Chojnacka, Rachel C. Murray

**Affiliations:** 1 Equine Department, Hartpury University, Gloucester GL19 3BE, UK; 2 Animal Health Trust, Newmarket CB8 7UU, UK; jahonka@poczta.onet.pl; 3 VetCT, Cambridge CB3 0FA, UK

**Keywords:** equine, orthopaedic injury, illness, eventing

## Abstract

**Simple Summary:**

The description of health patterns of eventing horses outside of veterinary clinics is limited. It is important to determine the relative impact of different health problems in this group of horses. The study aimed to describe the prevalence and patterns of lameness/illness in registered British eventing horses in 2018 by use of an online survey. Data were collated and descriptive analyses undertaken. In total, 1677 surveys were completed, and 49.4% reported a previous lameness/musculoskeletal problem, with 26% in the previous six months. The most frequently reported musculoskeletal problems were to the foot (421), joints (382), wounds (340), back (333), ligament (205), tendon (213), bones (140) and muscles (135). The most frequent non-musculoskeletal problems were to the skin (183), gastric ulceration (173), colic (145) and infection (88). Injuries to the sole of the foot/muscles/tendons were most frequently sustained in competition, suspensory ligament/splint bone/stifle/hock injuries were more frequently sustained in training and foot abscesses/penetration injuries at rest. Horses with deep digital flexor tendon injuries were out of training for the most amount of time (>12 months) and horses with hock/sole bruising/foot abscesses for the least amount of time (<2 weeks). A greater understanding of health problems in eventing horses could be useful for veterinarians working with them.

**Abstract:**

There has been little investigation into the health patterns of the eventing horse population outside veterinary clinics. To target health problems in the eventing horse population, it is important to determine the relative impact of different health problems. The objectives were to describe the prevalence and patterns of lameness/illness in registered British eventing horses. An online survey was released for all horses registered with British eventing in 2018. Data were collated and descriptive analyses undertaken. A total of 1677 surveys were completed, among which 49.4% reported a previous lameness/musculoskeletal problem, 26% being in the previous six months. The most frequently reported musculoskeletal problems were in the foot (421), joints (382), wounds (340), back (333), ligament (205), tendon (213), bones (140) and muscles (135). The most frequent non-musculoskeletal problems related to the skin (183), gastric ulceration (173), colic (145) and infection (88). Injuries to the hoof sole/muscles/tendons were most frequently sustained in competition, suspensory ligament/splint bone/stifle/tarsal injuries were more frequently sustained in training and abscess/foot penetration at rest. DDFT injuries were most frequently out of training for >12 months, SDFT/stifle/suspensory ligament for <12 months, tendon sheath/splint bone for <3 months and tarsal/sole bruising/abscess for <2 weeks. A greater understanding of injuries/illnesses frequently sustained could be useful for veterinarians working with event horses.

## 1. Introduction

In order to target health problems in the eventing horse population and reduce the risk of injury, it is important to determine the relative impact of different health problems. To date, there have been very few investigations describing the health patterns of the eventing horse population. Clinical reports suggest the foot, the digital flexor tendons of the forelimbs, the suspensory ligament and the patella are frequently injured sites in event horses [1,2]. A study describing injury risk in horses competing in different disciplines indicated that elite eventers had a high risk of injury to the forelimb superficial digital flexor tendon (SDFT) and a low risk of injury to the foot and tarsus compared to general-purpose horses [3]. Barstow and Dyson [4] reported that sacroiliac pain was most frequently diagnosed in horses working primarily in dressage (35%), general purpose (28%) and eventing (25%), and in 85% of cases, the horses had pain or lameness elsewhere. However, these data were from a referral clinic caseload, so less complex injuries that are able to be diagnosed in first-opinion practices would not have been reported. 

A survey-based study describing injuries in low-level event horses in the United States of America showed that ligament tear/desmitis (mainly suspensory ligament), joint disease/arthritis (mainly the stifle), foot/hoof injury and muscle strain/damage were most frequently reported [5]. Tendon and back problems were also reported but at much smaller percentages. Eighteen percent of the reported injuries occurred at a competition and the remainder occurred either during training or at rest. A description of injuries sustained by a range of upper and lower-level event horses during training and competition in the United Kingdom reported that during the cross-country phase of competition, the most common injuries reported were to the carpus and stifle [6]. SDFT injuries were significantly more common during elite-level international three-day competitions compared to low-level national one-day competitions. During training, injuries to the SDFT and suspensory ligament were most frequently reported. In elite-level Dutch event horses and ponies, reasons for withdrawal from the European Eventing Championships for ponies (2010) and horses (2011) were reported to be musculoskeletal in 45%, with tendon injuries being the most frequent, followed by fetlock joint injury [7]. However, there are limited numbers of horses included in these studies. 

Although the prevalence of non-musculoskeletal problems has been reported in horses doing some other sports, to the authors’ knowledge there are, to date, no studies describing their prevalence in eventing horses. There is also no description of how veterinary problems were associated with the duration of the period out of work or out of competition. 

This study aimed to describe the prevalence and patterns of veterinary problems, including musculoskeletal injury and illness in registered British eventing horses. The objectives were to describe (1) patterns of injuries and illness in British eventing horses; (2) which injuries and illness occurred most frequently during competition, training and rest; and (3) how long it took horses to return to work following frequently diagnosed injuries and illness.

## 2. Materials and Methods

The study was approved by the Ethical Review Committee of the Animal Health Trust (project number: AHT 54-2017). 

A survey was piloted among 30 horse-owners and amendments were made in response to their feedback. A 35-question online survey (SurveyMonkey, San Mateo, CA, USA) was released for all horses registered with British Eventing in the 2018 season, with a prize draw promotion. The survey was distributed through social media and e-newsletters sent by British Eventing. Riders with multiple horses were invited to complete one survey per horse. 

The survey was divided into seven sections: (1) rider information; (2) horse details and profile; (3) horse health, injury and illness; (4) horse management; (5) horse training and work-related information; (6) training surfaces; and (7) competition information. The variables collected and reported in this study are listed in Table 1. The survey consisted of open and closed questions. Free text boxes were provided for the “diagnosis” and “treatment” questions. At the end of data collection, the different diagnoses and treatments were grouped manually for analysis. For the questions on the reported time of onset of a problem, respondents had the choice of competition, training and rest. For the questions on the reported time out of work (training and competition), respondents had the choice of less than 2 weeks, less than 1 month, less than 3 months, less than 6 months, less than 12 months and more than 12 months. A copy of the survey is available in Supplementary Information S1.

Data were downloaded from SurveyMonkey into an Excel spreadsheet and descriptive analyses were undertaken. Surveys where only Sections 1 and 2 were completed were excluded from the analysis. Descriptive statistics were performed for each dataset using a statistical software package (Analyse-It, Leeds, UK). Data from sections 2 (horse detail and profile) and 3 (horse health, injury and illness) are reported in this study.

## 3. Results

There were 1677 responses collected out of 11,205 horses registered with BE in 2018, resulting in a 15.0% response rate.

### 3.1. Horse Features

Horse gender was most frequently gelding (68.5%), breed was most frequently Irish Sport Horse (37.1%), height was most frequently in the 15.3–16.2 hh category (50.5%) and age was most frequently reported as 9 years old (13.3%). The majority of the horses had been competing for one or two years (22.8% and 20.3%, respectively). The “current level of training” most frequently reported was BE100 (23.3%), which was also the “highest level of competition ever” most frequently reported (25.1%). The “highest level of competition that season” most frequently reported was BE90 (27%); however, data were collected for horses competing at all levels. BE90 and BE100 were the levels “most frequently competed at” in 2018 (45.4% and 40.8%, respectively). The majority of horses competed between seven and nine times a year (31.5%), competed twice a month (43.2%) and/or had two weeks between eventing competitions (36.8%). Furthermore, 37.4% of horses competed in dressage once a month, 42.8% in showjumping once a month and 9.7% undertook team chasing/hunter trials once a month. Other reported activities included arena eventing, combined training, endurance and pony club. Most horses (51.4%) had not participated in a previous different sport (Table 2).

### 3.2. Lameness and Musculoskeletal Problems

A total of 1994 musculoskeletal problems were reported. A previous lameness or musculoskeletal problem was reported by 49.4% of respondents. Of these, 26% reported one or more episodes in the previous 6 months. The most frequently reported musculoskeletal structures affected in order of frequency were foot (*n* = 421), joint (*n* = 382), spinal (*n* = 346), ligament (*n* = 205), tendon (*n* = 213), bones (*n* = 140) and muscles (*n* = 135), with neurological (*n* = 10) problems least frequently reported. 

**Figure 1:**
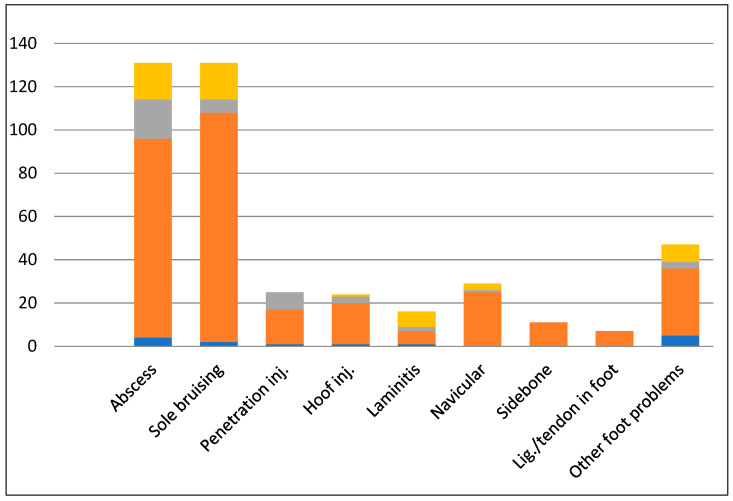
Distribution of 421 foot injuries/problems reported in British eventing horses. The orange area represents the front feet, the grey area represents the hind feet and the yellow area represents the front and hind feet. The blue area on the bars represents missing information on which limb was affected. Inj = injury, lig = ligament.

Figure 1 shows the types of foot problems reported. Abscesses (*n* = 131) and sole bruising (*n* = 131) were most frequently reported. All problems reported were more frequently observed in the forelimb than the hindlimb. 

**Figure 2:**
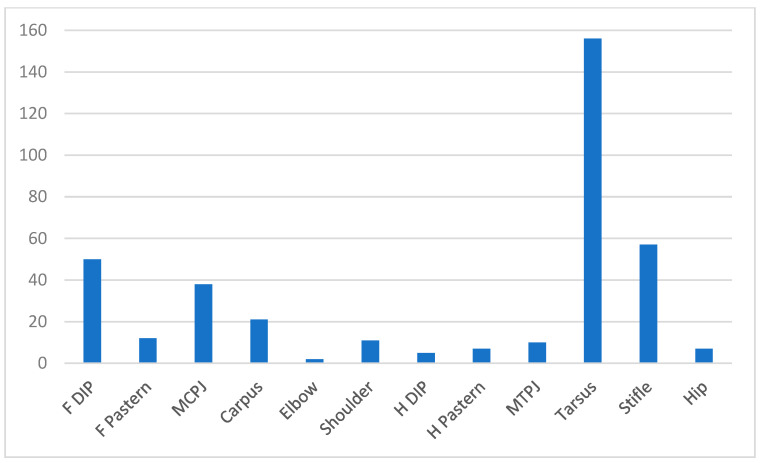
Distribution of 382 joint injuries reported in British eventing horses. F = forelimb, H = hindlimb, DIP = distal interphalangeal joint, MCPJ = metacarpophalangeal joint, MTPJ = metatarsophalangeal joint.

Figure 2 shows the distribution of joint problems. The tarsus had the most injuries (*n* = 156), stifle (*n* = 57), distal interphalangeal joint (50 forelimb; 5 hindlimb), fetlock (38 forelimb; 10 hindlimb), carpus (*n* = 21), pastern (12 forelimb; 7 hindlimb), shoulder (*n* = 11), hip (*n* = 7) and elbow (*n* = 2). The most likely type of problem was arthritis (*n* = 244), followed by sprain/ligament problem (*n* = 33) and fracture (*n* = 23). 

**Figure 3:**
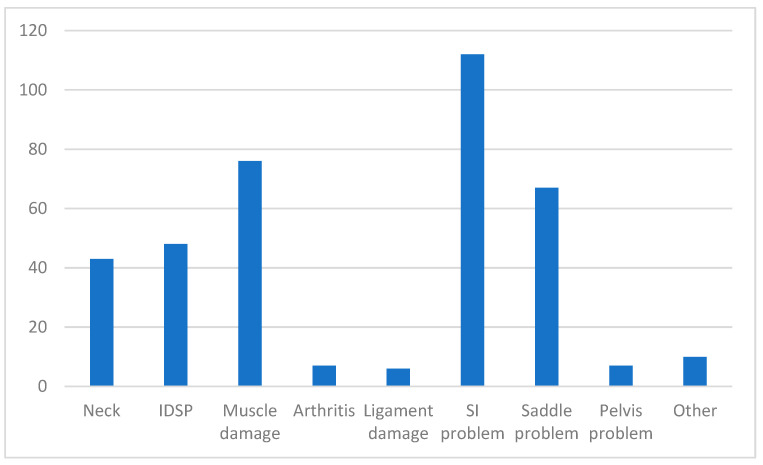
Distribution of 346 spinal injuries/problems reported in British eventing horses. SI = sacroiliac. IDSP = impinging dorsal spinous processes.

Of spinal problems reported, sacroiliac problems (*n* = 112) were the most frequent, followed by muscle damage (*n* = 76), saddle problem (*n* = 67) and impinging dorsal spinous processes (*n* = 48). Neck problems were reported in 43 horses, with neck muscle strain being most frequently reported (19/43), followed by neck trauma (8/43) and arthritis (7/43). Very few horses were reported as having a diagnosis of arthritis, ligament damage or pelvic problems (Figure 3). 

**Figure 4:**
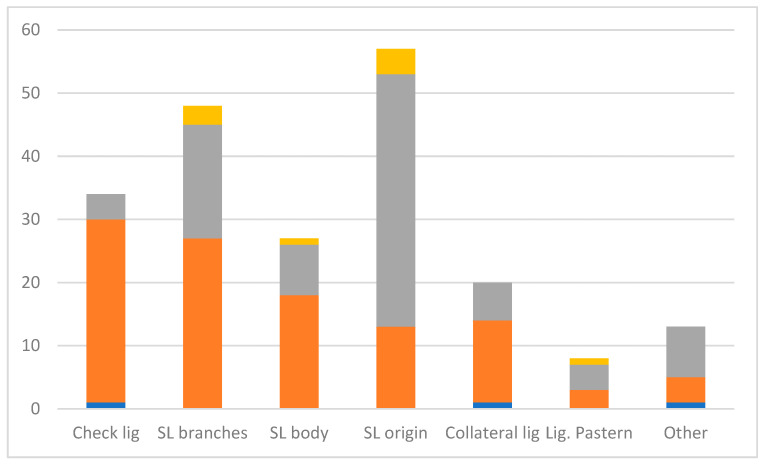
Distribution of 205 ligament injuries/problems reported in British eventing horses. The orange area represents the forelimb, the grey area represents the hindlimb and the yellow area represents both. The blue area on the bars represents missing information on which limb was affected. Lig = ligament, SL = suspensory ligament.

A total of 205 ligament problems were reported (Figure 4). Suspensory ligament injury was the most likely problem reported, with problems at the origin (*n* = 57) most frequently reported, followed by the branches (*n* = 48) and the body (*n* = 27). Other ligament problems reported were accessory ligament of the deep digital flexor tendon (check ligament) (*n* = 34), collateral ligament (*n* = 20), pastern (*n* = 8) and other (*n* = 13). Ligament injuries were more frequently reported in the forelimb, except for problems at the suspensory ligament origin, which were more likely in the hindlimb (44/57). 

**Figure 5:**
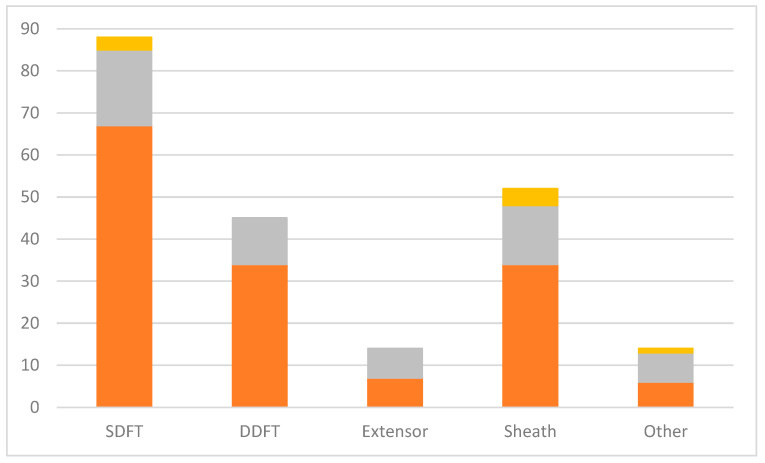
Distribution of 213 tendon injuries/problems reported in British eventing horses. The orange area on the bars represents the forelimb, the grey area represents the hindlimb and the yellow area represents both. SDFT = superficial digital flexor tendon, DDFT = deep digital flexor tendon, Sheath = tendon sheath, Other = other tendon.

In total, 213 injuries to the tendons were reported (Figure 5). SDFT (88/213) was the most frequent tendon injured. Injuries to the forelimb were more frequent than hindlimb for the SDFT (67/88), deep digital flexor tendon (DDFT) (34/45) and tendon sheath (34/52). Extensor tendon injuries were reported equally in the forelimbs and hindlimbs (Figure 5). The most reported types of injuries were core lesions (17/88) in the SDFT, tears in the DDFT (9/45) and tendon sheath infection/inflammation (12/52) or laceration (9/52). 

**Figure 6:**
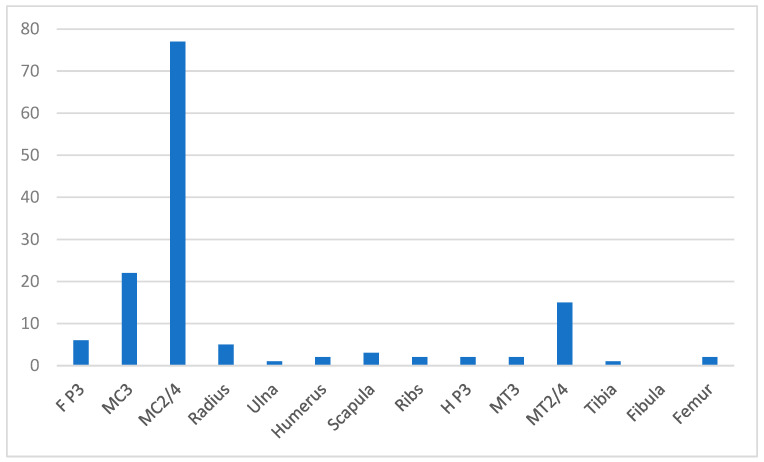
Distribution of 140 bone injuries/problems reported in British eventing horses. F P3 = forelimb distal phalanx, MC3 = 3rd metacarpal bone, MC2/4 = 2nd and 4th metacarpal bones, H P3 = hind distal phalanx, MT3 = 3rd metatarsal bone, MT2/4 = 2nd and 4th metatarsal bones.

A total of 140 bone injuries were reported (Figure 6). Splint bones were the most commonly reported location of bone injury with 77 forelimb and 15 hindlimb injuries. Problems with the third metacarpal bone (22/140), third metatarsal bone (2/140), distal phalanx (6/140) and radius (5/140) were also reported, with a few other locations reported infrequently. The most frequent problem type reported was a splint (89), followed by fracture/stress fracture (33) and bone bruising (10).

In total, 135 horses were reported to have muscle problems, with muscle strains being the most prevalent (47/135), followed by tying-up (45/135). Neurological problems were reported 10 times, with shivers being the most reported condition (5/10). 

### 3.3. Non-Musculoskeletal Problems (Table 3)

A total of 1400 non-musculoskeletal problems were reported, with the most frequent problems being skin wounds (*n* = 340), skin problems (n = 183), gastric ulceration (*n* = 173), colic (*n* = 145) and infection (*n* = 88). Lacerations/cuts were the most frequently reported skin wounds (66/340, 19%). Equine pastern dermatitis was the most frequently reported skin problem (31/183; 17%). Grade 2 stomach ulcers were most frequently reported (9/173; 5%). Impaction colic was most frequently reported (9/145; 6%). General virus was the most frequently reported infection problem (18/88; 20%).

### 3.4. Reported Time of Onset: Competition, Training or Rest (Table 4)

Foot abscesses and laminitis most frequently occurred in training (40% and 35%, respectively) or rest (47% and 53%, respectively), while sole bruising and navicular problems most frequently occurred in competition (34% and 32%, respectively) or training (38% and 46%, respectively). Hoof injuries most frequently occurred during training (33%) and nearly all penetrations were reported at rest (64%).

Metacarpophalangeal joint, forelimb distal interphalangeal joint, tarsal and stifle joint pathology most frequently occurred during training (46%, 38%, 48% and 39%, respectively). Sacroiliac, back muscle damage and impinging dorsal spinous processes also most frequently occurred during training (51%, 45% and 54%, respectively). 

SDFT, DDFT and suspensory ligament origin injuries most frequently occurred in competition (40%, 39% and 44%, respectively). Tendon sheath and check ligament injuries most frequently occurred in training (32% and 32%, respectively) and rest (32% and 32%, respectively), while suspensory ligament branch and body injuries most frequently occurred in training (35% and 41%, respectively).

For bone injuries, it was reported that in the forelimb, splint bone and metacarpal bone problems most frequently occurred in training (55% and 50%, respectively), whereas most hind splint bone problems most frequently occurred at rest (53%). 

Muscle strains most frequently occurred during competition and training (34% and 34%, respectively), bruises during competition (19%) and tying-up primarily during training (55%). Skin wounds most frequently occurred during rest (49%). Gastric ulceration most frequently occurred during competition (54%) and colic most frequently occurred during rest (59%).

### 3.5. Report Time Out of Work Following Injury or Illness (Table 5)

Reported time out of work for the majority of horses with a foot abscess or sole bruising was less than 2 weeks (59% and 45%, respectively), while a foot penetration, hoof injury or laminitis was reported to take longer in more horses (19/25, 18/24 and 13/16 took up to than 3 months). Navicular problems ranged in time out of work reasonably evenly across the options, with slightly more reported to have 1–3 months off.

Most metacarpophalangeal joint and tarsal injuries were reported to be off work for less than 2 weeks, while stifle problems were off work for longer, with 59% being off for up to 6 months. Forelimb distal interphalangeal joint injuries were more frequently reported to be off work for up to 6 months (66%).

Sacroiliac problems were most frequently reported to be off work for up to 3 months (22%). Horses with muscle damage or saddle fit issues were most likely to be off work for less than 2 weeks (34% and 60%, respectively), with nearly all being back to work by 3 months. 

Only 8% of SDFT injuries reported had time off of less than 1 month, with the majority taking much longer: 22% taking 1–3 months, 26% taking 3–6 months, 16% taking 6–12 months and 23% taking more than 12 months. A greater proportion of DDFT injuries were reported to take longer to recover, with only 9% taking less than 3 months off, and 24% taking 3–6 months, 22% taking 6–12 months and 31% taking more than 12 months. In contrast, extensor tendon injuries were more likely to recover quicker, with 21% taking less than 6 months off. Check ligament injuries took less time off, with 53% taking less than 3 months and 15% taking more than 6 months. For suspensory ligament branch problems, the majority reported 1–3 (23%), 3–6 (25%) or 6–12 (21%) months off, which was a pattern also seen in the suspensory ligament body. The suspensory ligament origin tended to take longer to return to work, with most taking 3–6 (25%) or 6–12 months (32%) or longer. It was reported that injuries to DDFT were most frequently out of training for more than 12 months (31%). SDFT problems most frequently resulted in less than 6 months out of training (30%). Stifle and suspensory ligament origin problems were out of training for less than 12 months (25% and 32%, respectively). Tendon sheath problems were out of training for less than 3 months (38%). 

Forelimb splint bone (67%) or third metacarpal problem (64%) most frequently took less than 3 months off work. Muscle bruises, strains, tying-up, skin wounds, gastric ulceration, colic, skin problems and infections (bacterial and viral) resulted in horses being most frequently off work for less than 2 weeks (60%, 44%, 48%, 60%, 43%, 73%, 62% and 42%, respectively).

## 4. Discussion

The results showed that injuries to the foot, joints, back, ligaments, tendons, bones and muscles were most frequently reported. Skin problems, gastric ulceration and colic were the most frequent non-musculoskeletal problems reported. The timing of injury occurrence varied depending on the type of injury and illness. The amount of time off work varied depending on the type of problem. 

The high proportion of foot/hoof injuries is not a surprising finding as it supports previous reports [1,2,5]. Abscesses and sole bruising, in equal numbers, were the most frequent foot problems reported. Abscesses tended to occur during training or at rest, whereas sole bruising tended to occur during competition or training. DeBowes and Yovich [8] indicated that foot tissue that is bruised can become infected and lead to abscess formation. This is a possible explanation as to why sole bruising tended to occur in competition/training and abscesses tended to be observed in training/rest if sole bruising sustained in competition potentially leads to subsequent abscess formation, potentially then noted at training/rest.

Previous studies have indicated that the distal interphalangeal joint of the forelimb, stifle and fetlocks are joints that are frequently injured [1,2,5,7], which is in accordance with findings from the current study. The frequency of occurrence of tarsal pain in the general population [9] may also be consistent with the finding in the current study that the tarsus was the joint most frequently reported as a problem. This contrasts with a previous study which indicated that elite eventing horses had a decreased risk of tarsal injury compared to general-purpose horses [3]. However, it is probable that the referral caseload used in the previous study by Murray et al. [3] may have skewed the results because tarsal pathology can successfully be diagnosed in first-opinion practice. 

Arthritis was the most frequently reported joint problem in the current study, which is in accordance with the findings of Caston and Burzette [5]. Arthritis is a common degenerative joint disease in horses [10,11]. It is therefore not surprising that it was a commonly reported joint condition. It is interesting to note that metacarpophalangeal and tarsal joint pathology resulted in the least time off work (less than 2 weeks), followed by forelimb distal interphalangeal joint injuries (less than 6 months) and stifle injuries requiring the most time off work (more than 6 months), potentially because the former may be more straightforward to diagnose and manage than stifle pain, which can be more challenging [12]. 

Studies have shown that sacroiliac pain can contribute to poor performance [13,14,15], and in British dressage horses, sacroiliac pain/problems were the third most frequent back problem reported [16]. In the current study, sacroiliac pain was the most frequently reported back problem. Barstow and Dyson [4] suggested that sacroiliac pain was more common in dressage horses than eventing horses (35% vs. 25%), but this could potentially be due to the caseload on which that study was based. Studies have shown that hindlimb lameness alters movement of the back [17,18,19], so it is possible that sacroiliac pain could develop due to gait alterations [20]. It is possible that the horses in the current study had concurrent hindlimb lameness or other pathology related to the back. 

Injuries to the origin of the hindlimb suspensory ligament were the most frequently reported ligament problem. In the current study, 84.3% of the horses were competing at the Novice level or lower and therefore considered non-elite. The dressage tests for the lower levels mainly consist of trot work. It is possible that these horses did not have sufficient muscular strength to prevent hyperextension of the metatarsophalangeal joint during the stance/loading phase of the stride, which would increase the load experienced by the suspensory ligament during trot [21] and cause injury. A previous study has suggested that an increase in tarsal and metatarsophalangeal joint mediolateral oscillation is related to poor muscular strength, resulting in instability of the distal limb [22].

Suspensory ligament origin injuries were mainly sustained in competition, although the difference between the number of training-reported injuries was small. Concurrent tarsal or sacroiliac pain and suspensory ligament origin injuries have been reported [4,23]. It is possible that the suspensory ligament origin injury was not the primary cause of lameness and manifested itself as a secondary cause observed during poor performance either at a competition or during training. Additionally, 65% of the horses took more than 6 months to return to work. This is in a similar region to previous studies showing that the time to return to work was influenced by the treatment. One study showed that with controlled walking and radial pressure wave therapy, horses returned to full work six months after diagnosis [24]. However, if platelet-rich plasma intralesional injections were used, horses could return to work after 12 weeks [25].

The forelimb SDFT was the most frequent tendon injured. This supports the findings of Murray et al. [3], who reported that elite eventers had a very high risk of injury to this tendon compared to general-purpose horses despite the current study investigating horses using the new eventing format whereas the older study reported on horses competing using the previous longer format eventing. SDFT injuries are also the most common cause of lameness in National Hunt racehorses, with a prevalence as high as 24% [26,27]. It has previously been shown that the forelimb SDFT is likely to experience high strains associated with landing after fences [28] and galloping at speed, which could predispose this structure to injury. 

SDFT injuries were reported to mainly occur during competition, and the majority of horses spent between 3 and 6 months off work, which is likely related to the severity of the lesion and the stages of healing [29]. Singer et al. [6] showed that tendon or ligament injuries were conditions suffered by Intermediate-level event horses that prevented those horses from competing. The same study also showed that the SDFT was the soft tissue most frequently injured and these injuries tended to manifest themselves during training. In the current study, we found that SDFT injuries were reported to most frequently occur during competition. This could be due to the timing of data collection. Singer et al. [6] collected information at events, whereas the current study collected information retrospectively. The clinical signs of SDFT injury are frequently swelling and heat on the palmar aspect of MCIII and these signs may not be observed until the day after the event, but could still have been attributed to an injury at the event by the rider/support team. A retrospective study would collect this information whereas a study collecting information at events would not unless the injury were so severe that it could be diagnosed by the competition treating veterinarian.

Injury to the forelimb splint bones was the most frequently reported bone injury, with splints or fractures being most frequently observed. This is in accordance with previous literature [30,31,32]. Injuries to the splint bones have been reported to generally result from external trauma [30,31]. It is possible that the injuries reported in the study were the result of the horse impacting an obstacle such as a fence, or through interference by another limb as injuries most frequently occurred in training (47/86). Caston and Burzette [5] reported that 4% of the injuries sustained by their study population of low-level eventing horses in the United States were to the splint bones. This is a similar occurrence to that of the current study (5%) where 84.3% of the population were considered to be competing at a non-elite level. 

A range of non-musculoskeletal problems were reported, which were generally consistent with those expected in a general population [33,34]. Skin wounds were the most frequent non-musculoskeletal problem reported, which were reported to occur most frequently at rest, and usually returned to work in less than 2 weeks, with lacerations/cuts being the most frequent diagnosis. Theoret et al. [33] showed that skin wounds were the third most frequently reported problem in the general horse population, with 58% of owners treating the wounds without consulting their veterinarian. It is possible that the majority of wounds reported in the current study were superficial, which could be consistent with the rapid healing time reported. Equine pastern dermatitis was the most frequently reported skin problem (31/183; 17%). This is similar to the 16% reported by Altermatt et al. [35] in Swiss warmblood horses but slightly higher than the 12.5% reported by Maksimović et al. [36] in a group of horses in Bosnia and Herzegovina.

Prevalences for gastric ulceration have been reported to vary by population and exercise program [37]. It has been reported at levels of 37–52%, 38–56% and 48% in Thoroughbred, Standardbred and endurance racehorses, respectively, when out of training. These increase up to 100%, 72–88% and 57–93%, respectively, when these same populations are in training [38,39,40,41,42,43,44,45,46,47]. The prevalence of gastric ulcers in a group of Canadian showjumpers has also been reported at levels of 72% [48]. In elite-level three-day eventing horses in the United States of America, ulceration was reported at a prevalence of 42.2% [49]. Various reasons have been reported as risk factors for gastric ulcers. The list includes exercising more than five days per week/increased workload [48,50,51], travel [52], stress [44,53] and feeding management [49], amongst other variables. The current study showed that gastric ulcers were most frequently reported during competition, resulting in the horses being off work for less than 2 weeks. It is possible that travelling to the event, the increase in workload and the stress of the competition, especially in young inexperienced horses, could lead to an increase in the occurrence of gastric ulceration. In Thoroughbred and Standardbred racehorses, gastric ulceration has been associated with a decrease in performance [54,55], so it is possible that a reduction in performance could have led respondents to have attributed gastric ulceration occurrence to have been during competition. The current study showed that Grade 2 was most common (9/173); however, the grade and location of the ulcers were not recorded by the majority of the study respondents (144/173). It is possible that most of the ulcers reported were low-grade, which could explain why these horses returned to work in less than 2 weeks. It is likely that high-grade gastric ulcers may have required more time off work.

Within the general equine population, the prevalence of colic varies from 3.5 to 11.1 colic episodes per 100 horses per year depending on the study [56,57,58,59,60] and is the most common cause for emergency out-of-hours veterinary callouts [61,62]. In the current study, colic most frequently occurred at rest, resulting in the horse being off work for less than 2 weeks. Early recognition and treatment of colic have been shown to improve the outcome for the horse [63]. The current study showed that impaction colic was most commonly recorded (9/145); however, the type of colic was not recorded by the majority of the study respondents (119/145). It is possible that most colic cases reported were mild, which could explain why these horses returned to work in less than 2 weeks. Respondents did not record how the colic was managed, but it is likely that surgical management would be associated with more time off work. 

General virus was the most frequently reported infection problem (18/88; 20%). Bacterial and viral respiratory infections are an important problem in the British racehorse population, with a prevalence of 80% [64]. Wood et al. [64] also indicated that the prevalence reduces with an increase in age. Eventing horses mix at competitions, as racehorses do at the racetrack, but are less likely to be stabled at a competition and in comparison are stabled for fewer hours. This, along with the fact that the eventing population is older than the racing population, could be a possible explanation for the difference in the reported level of infection problems. 

### 4.1. Limitations

The 15% response rate was disappointing, but the range of respondents was a good reflection of the pattern of membership. The main limitation of our study is recall bias in the way the data were collected. There may have also been unreported injuries or a lower rate of injury due to a lack of responses, and in some cases, a specific lameness diagnosis was not given. Another limitation is that these data are limited to the United Kingdom’s eventing population during one season. Data from other countries and seasons may differ.

### 4.2. Further Work

In other sports, including dressage, showjumping, endurance and racing, the investigation of risk factors for injury has identified management and training risk factors that could potentially be modified [16,65,66,67,68,69,70,71,72,73,74,75]. There may be aspects of training or management of eventing horses that could be modified to reduce injury risk, which will be important to investigate.

## 5. Conclusions

A greater understanding of injuries and illnesses frequently sustained could be useful for veterinarians working with event horses. When evaluating event horses, foot, joint and back problems should be considered likely, in addition to tendon and ligament problems. 

## Figures and Tables

**Table 1:** Overview of the information collected in the British eventing horse survey that is described in this study.

Section	Specific Variable Questioned
Horse details and profile	Gender Breed Height Age Years competing in British Eventing competitions Current level of training Highest level of competition ever Highest level of competition this season All levels competed at this season Frequency of competing Number of weeks between events Frequency of competing in other disciplines Previous occupation
Horse injury and illness	System affected and type Tendon problem: location, diagnosis, treatment Ligament problem: location, diagnosis, treatment Foot problem: location, diagnosis, treatment Bone problem: location, diagnosis, treatment Joint problem: location, diagnosis, treatment Muscle problem: location, diagnosis, treatment Back problem: diagnosis, treatment Neck problem: diagnosis, treatment Neurological problem: diagnosis, treatment Other types of ill health and the activity the horse was doing when symptoms started How many episodes of lameness/injury in last 2 years How many episodes of ill health in last 2 years

**Table 2:** Horse profile summary for the 1677 study respondents. Information on gender, breed, height, age, years competing, current training level, highest level of competition ever, highest level of competition this season and all levels competed this season are described in this table. BE = British Eventing; FEI = Fédération Equestre Internationale; BE80 (T) = introductory level, maximum height of the show jumping fences are 85 cm and 80 cm for the cross-country fences; BE90 = maximum height of the show jumping fences are 95 cm and 90 cm for the cross-country fences; BE100 = maximum height of the show jumping fences are 105 cm and 100 cm for the cross-country fences: BE105 = maximum height of the show jumping fences are 110 cm and 105 cm for the cross-country fences; Novice = maximum height of the show jumping fences are 115 cm and 110 cm for the cross-country fences; Intermediate = maximum height of the show jumping fences are 125 cm and 115 cm for the cross-country fences; Advanced = maximum height of the show jumping fences are 130 cm and 120 cm for the cross-country fences; FEI* = equivalent to Novice, and FEI2* since 2019; FEI** = equivalent to Intermediate, and FEI3* since 2019; FEI*** = equivalent to Advanced, and FEI4* since 2019; FEI**** = the highest level of eventing, offered at six competitions around the world, and equivalent to FEI5* since 2019.

Variable		N
Gender	Mare	510
	Gelding	1116
	Stallion	4
Breed	Warmblood	307
	Thoroughbred	150
	Thoroughbred cross	240
	Irish Sport Horse	605
	Pony	101
	Pony cross	33
	Other	195
Height (hands high)	13.3–14.2	121
	14.3–15.2	283
	15.3–16.2	821
	16.3–17.2	378
	17.3–18.2	23
Age (years)	4	1
	5	36
	6	95
	7	153
	8	183
	9	215
	10	213
	11	161
	12	143
	13	121
	14	93
	15	60
	16	54
	17	32
	More than 17	62
Years competing	1	363
	2	323
	3	270
	4	201
	5	139
	6	92
	7	62
	8	48
	9	30
	10	25
	11	11
	12	9
	13 and more	17
Current training level	BE80 (T)	140
	BE90	366
	BE100	388
	BE105	114
	Novice	346
	Intermediate	135
	Advanced	35
	FEI*	41
	FEI**	30
	FEI***	54
	FEI****	18
Highest level of competition ever	BE80 (T)	171
	BE90	378
	BE100	418
	BE105	51
	Novice	267
	Intermediate	99
	Advanced	23
	FEI*	104
	FEI**	86
	FEI***	41
	FEI****	27
Highest level of competition this season	BE80 (T)	256
	BE90	438
	BE100	415
	BE105	42
	Novice	215
	Intermediate	70
	Advanced	15
	FEI*	70
	FEI**	43
	FEI***	36
	FEI****	22
Levels competed at this season	BE80 (T)	422
	BE90	722
	BE100	648
	BE105	107
	Novice	398
	Intermediate	172
	Advanced	45
	FEI*	137
	FEI**	81
	FEI***	44
	FEI****	15

**Table 3:** Summary of the most frequently reported non-musculoskeletal problems (*n* = 1400).

Problem	*n*
Heart	9
Equine asthma	64
Upper airway disorders	36
Lung problems	6
Infection (viral and bacterial)	88
Colic	145
Gastric ulceration	173
Diarrhoea	46
Inflammatory bowel disease	5
Metabolic problems	9
Reproductive problems	7
Urinary	4
Skin problems	183
Skin wounds	340
Head shaking	32
Head trauma	15
Sinus problems	11
Eye problems	74
Ear problems	6
Lip damage	35
Tongue damage	10
Tooth problems	71
Other	31

**Table 4:** Summary of whether the most frequently reported musculoskeletal injuries (*n* = 1994) and non-musculoskeletal problems (N = 1400) were reported to be sustained at competition, training or rest. MCPJ = metacarpophalangeal joint, F DIP = forelimb distal interphalangeal joint, SI = sacroiliac, IDSP = impinging dorsal spinous processes, SDFT = superficial digital flexor tendon, DDFT = deep digital flexor tendon, SL = suspensory ligament, MCII and IV = forelimb splint bones, MCIII = forelimb cannon bone, MTII and IV = hindlimb splint bones.

Condition/Structure	Competition (*n*)	Training (*n*)	Rest (*n*)	Unspecified (*n*)
Foot abscess	10	59	68	9
Laminitis	0	6	9	2
Sole bruising	52	59	25	18
Navicular problems	12	17	6	2
Hoof injury	5	8	6	5
Hoof penetration injury	2	4	16	3
MCPJ	15	22	6	5
F DIP	18	19	5	8
Tarsus	52	101	41	17
Stifle	22	27	14	7
SI	38	57	15	2
Back muscle damage	17	24	12	0
IDSP	15	35	13	2
SDFT	37	30	22	3
DDFT	19	14	13	3
Extensor tendon	5	3	3	3
SL origin	32	29	5	7
Tendon sheath	16	18	18	5
Check ligament	10	11	11	2
SL origin	32	29	5	7
SL branch	17	19	12	7
SL body	10	14	8	2
MCII and IV	24	47	8	7
MCIII	6	11	4	1
MTII and IV	2	4	9	2
Muscle strain	15	15	5	9
Muscle bruise	5	2	1	18
Tying-up	14	31	7	4
Skin wounds	98	74	165	3
Gastric ulceration	94	57	19	3
Colic	21	37	86	1

**Table 5:** Summary of the time out of work reported for different musculoskeletal injuries (*n* = 1994) and non-musculoskeletal problems (N = 1400). MCPJ = metacarpophalangeal joint, F DIP = forelimb distal interphalangeal joint, SI = sacroiliac, IDSP = impinging dorsal spinous processes, SDFT = superficial digital flexor tendon, DDFT = deep digital flexor tendon, SL = suspensory ligament, MCII and IV = forelimb splint bones, MCIII = forelimb cannon bone, MTII and IV = hindlimb splint bones.

Condition/Structure	<2 Weeks (%)	<1 Month (%)	<3 Months (%)	<6 Months (%)	<12 Months (%)	>12 Months (%)	Missing (%)
Foot abscess	59	24	10	1	0	0	6
Laminitis	38	25	19	0	6	12	0
Sole bruising	45	24	18	4	1	0	8
Navicular problems	7	10	28	21	17	14	3
Hoof injury	33	21	21	4	0	4	17
Hoof penetration injury	28	32	16	4	4	4	12
MCPJ	33	13	13	13	5	5	18
F DIP	16	18	19	19	11	16	1
Tarsus	37	21	19	6	7	4	6
Stifle	19	14	16	16	25	4	6
SI	32	19	21	10	6	10	2
Back muscle damage	35	22	21	8	4	1	9
IDSP	19	13	19	29	8	10	2
Saddle problems	55	24	10	4	0	0	7
SDFT	3	5	22	26	16	23	5
DDFT	0	2	7	24	22	31	14
Extensor tendon	7	7	22	22	14	7	21
Tendon sheath	12	13	38	10	6	10	11
Check ligament	3	9	41	24	12	3	8
SL origin	0	7	21	25	32	9	6
SL branch	4	8	23	25	21	10	9
SL body	4	11	33	19	26	4	3
MCII and IV	29	16	31	9	5	0	10
MCIII	23	14	36	23	0	0	4
MTII and IV	27	0	27	20	13	0	13
Muscle Strain	36	30	11	6	0	2	15
Muscle Bruise	46	23	8	4	0	0	19
Tying-up	47	27	11	7	0	0	8
Skin wounds	60	20	9	4	1	0	6
Gastric ulceration	43	17	21	6	2	1	10
Colic	73	8	4	7	2	0	6
Skin problems	62	16	9	0	2	2	9
Infections (viral and bacterial)	42	24	25	6	0	1	2

## Data Availability

Data are unavailable due to privacy or ethical restrictions.

## Supplementary Materials

The following supporting information can be downloaded at: https://www.mdpi.com/article/10.3390/ani14182667/s1, Supplementary S1: Copy of the survey that was completed.
