# Supplementary material for: Musculoskeletal Injury and Illness Patterns in British Eventing Horses: A Descriptive Study
Source: Animals (Basel). 2024 Sep 13;14(18):2667. doi: 10.3390/ani14182667 (PMC11429326; doi:10.3390/ani14182667)
Supplement: Supplementary file 1 [file animals-14-02667-s001.zip › animals-3195262-supplementary.pdf]

The Animal Health Trust (AHT) is working with BE and the BEF to investigate the risk factors for injury and falls in competition, in British Eventing horses.

Injuries can be a major problem so we need to know how to prevent them. By providing information, whatever level you are and whether your horse is lame or sound, you will help these answers.

We need YOUR help to improve the future health and performance of Event horses.

Please complete one questionnaire for each horse registered with BE. All information provided is STRICTLY CONFIDENTIAL.
The questionnaire will take you approximately 30 minutes to complete.
You can also complete the questionnaire online at:
https://be-questionnaire-2018.onlinesurveys.ac.uk/injuries-and-falls-in-british-eventing-horses

PRIZE DRAW - To be entered in the prize draw please supply your email address at the end of the questionnaire; we will only contact you if you are a winner.

Completion of the questionnaire indicates your consent to participate in this study. Your personal information will be held and used in accordance with the Data Protection Act 1998 and will not be disclosed to any unauthorised person or body. Only the study investigators will have access to the data collected. The data collected will be securely stored at the AHT. Anonymised results may be written in a report for publication and presented at research conferences or meetings. This study has received ethical approval (Reference Number: AHT54-2017).

SECTION 1: RIDER PROFILE

Please provide competition rider details (circle as appropriate):

1. Height: ____ cm / ft
2. Approximate weight: ____ kg / st
3. Age: ____ years
4. Gender: Female Male Prefer not to say
5. Years registered with BE: ____ years
6. Highest level of BE competition:

SECTION 2: HORSE DETAILS

1. What sex is the horse? (Please select one only)

Mare
Gelding
Stallion

2. What breed is the horse registered as?

Warmblood
Thoroughbred
Thoroughbred cross
Irish Sport Horse
Pony
Pony cross
Other

3. What height is the horse? (Please select one only)

13.2 or less
13.3-14.2hh
14.3-15.2hh
15.3-16.2hh
16.3-17.2hh
17.3-18.2hh
More than 18.2hh

4. What age is the horse? _____ years

5. Years of competing in BE competitions: _____ years

6. Please provide information about your horse's level of training and competition:
(Please select one only)

**Current** level of training:	BE80(T)<br>BE90<br>BE100<br>BE105	Novice<br>Intermediate<br>Advanced	FEI*<br>FEI**<br>FEI***<br>FEI****
**Highest** level of competition **ever**:	BE80(T)<br>BE90<br>BE100<br>BE105	Novice<br>Intermediate<br>Advanced	FEI*<br>FEI**<br>FEI***<br>FEI****
**Highest** level of competition **this season**:	BE80(T)<br>BE90<br>BE100<br>BE105	Novice<br>Intermediate<br>Advanced	FEI*<br>FEI**<br>FEI***<br>FEI****

7. At what levels has the horse competed this season (2018)? (Please select **all** levels)

BE80(T)	Novice	FEI*
BE90	Intermediate	FEI**
BE100	Advanced	FEI***
BE105		FEI****

8. In the <u>last two years</u>, how frequently did the horse compete in Eventing during the season ('compete' refers to the total number of Events attended)?

	Per year		Per month	
On average, number of times:	Less than 1<br>1-3<br>4-6<br>7-9	10-12<br>13-15<br>more than 15	Less than 1<br>1<br>2	3<br>4

9. On average, how many weeks do you leave between BE events?

1 week
2 weeks
3 weeks
4 weeks
5 weeks
6 weeks
7 weeks
8 weeks
More than 8 weeks

10. On average, how many times per month does the horse compete in other disciplines? (Please circle one)

Dressage	None	Less than 1	1	2	3	4	More than 4
Show jumping	None	Less than 1	1	2	3	4	More than 4
Team chasing/ hunter trials	None	Less than 1	1	2	3	4	More than 4
Hunting	None	Less than 1	1	2	3	4	More than 4
Other, please specify:	None	Less than 1	1	2	3	4	More than 4

11. What (if any) was the horse's previous occupation? (Please select all relevant options)

Not applicable
Hunting
Showjumping
Dressage
Jump racing
Flat racing
Pony Club/ Riding Club
Pleasure
Western
Endurance
Other (please specify) __________

SECTION 3: YOUR HORSE'S HEALTH

1. Has this horse experienced lameness or any other musculoskeletal/orthopaedic problems? Yes No

2. If YES, what musculoskeletal/orthopaedic conditions has this horse experienced? (Please select all the relevant options) If NO, go to page 9.

a) Tendon problem

Condition	Limb			When sustained			Time off work	Diagnosis (if known)	Comments
	Front	Hind	Both	Competition	Training	Rest			
Superficial digital flexor tendon									
Deep digital flexor tendon									
Extensor tendon									
Tendon sheath problem									
Other (please specify)									

b) Ligament problem

Condition	Limb			When sustained			Time off work	Diagnosis (if known)	Comments
	Front	Hind	Both	Competition	Training	Rest			
Check ligament									
Suspensory ligament - Branches									
Suspensory ligament - Body									
Suspensory ligament - Origin (proximal suspensory desmitis)									
Collateral ligaments (specify joint, eg coffin, pastern, fetlock)									
Ligaments in pastern area									
Ligaments in the foot									
Other (please specify)									

c) Foot problem

Condition	Limb			When sustained			Time off work	Diagnosis (if known)	Comments
	Front	Hind	Both	Competition	Training	Rest			
Abscess									
Sole bruising									
Penetration injury									
Hoof injury									
Laminitis									
Navicular problems									
Sidebone									
Coffin joint problem									
Ligament or tendon in foot									
Other (please specify)									

d) Bone problem

Bone	Condition							When sustained			Time off work	Diagnosis (if known)	Comments
	Bruise	Cyst	Fracture	Infection	Splint	Repetitive overload injury/stress fracture	Other	Competition	Training	Rest			
Front cannon bone													
Front splint bone													
Hind cannon bone													
Hind splint bone													
Radius													
Ulna													
Humerus													
Scapula (shoulder blade)													
Tibia													
Fibula													
Femur													
Ribs													

e) Joint problem

Joint	Condition													When sustained			Time off work	Diagnosis (if known)	Comments
	Bruise	Cyst	Fracture	Inflammation	Arthritis	Osteochondrosis dissecans (OCD)	Cartilage damage	Ligament problem	Sprain	Heat	Penetration injury	Chip fracture	Other	Competition	Training	Rest			
Coffin joint front																			
Coffin joint hind																			
Fetlock joint front																			
Fetlock joint hind																			
Pastern joint front																			
Pastern joint hind																			
Carpus/ knee																			
Shoulder																			
Elbow																			
Hock																			
Stifle																			
Hip																			

f) Muscle problem

Condition	Limb			When sustained			Time off work	Diagnosis (if known)	Comments
	Front	Hind	Both	Competition	Training	Rest			
Bruise									
Infection									
Strain									
Tying up									
Other (please specify)									

g) Back problem

Condition	When sustained			Time off work	Diagnosis (if known)	Comments
	Competition	Training	Rest			
Kissing spines						
Muscle damage						
Arthritis						
Ligament damage						
Sacroiliac problem						
Saddle problem						
Other (please specify)						

h) Neck problem

Condition	When sustained			Time off work	Diagnosis (if known)	Comments
	Competition	Training	Rest			
Arthritis						
Fracture						
Ligament damage						
Ligament strain						
Muscle strain						
Neck trauma						
Wobbler						
Other						

i) Neurological problem

Condition	When sustained			Time off work	Diagnosis (if known)	Comments
	Competition	Training	Rest			
Ear problem						
Head trauma						
Neck damage						
Neck trauma						
Seizure						
Shivers						
Stringhalt						
Wobbler						
Other						

3. In which of the following categories has the horse experienced ill health and what activity was the horse doing when the symptoms started? (Please select all the relevant options)

Condition		Competition	Training	Rest	Diagnosis (if known)	Time off work
Heart/Cardiac disorders						
Respiratory	Equine asthma /COPD/RAO					
	Upper airways disorders					
	Lung problems					
	Infection (viral/bacterial)					
Gastrointestinal	Colic					
	Stomach ulcers					
	Diarrhoea					
	Inflammatory Bowel Disease					
Metabolic (e.g. EMS, cushings)						
Reproductive						
Urinary						
Skin disorders						
Wounds						
Head shaking						
Head trauma						
Sinus problem						
Eye problem						
Ear problem						
Lip damage						
Tongue damage						
Tooth problem						
Other (please specify in diagnosis)						

4. Approximately how many episodes of lameness/injury has the horse experienced in each of the following time periods?

	Last 6 months	6-12 months ago	1-2 years ago	More than 2 years
Lameness/ injury				
Ill health problems				

SECTION 4: HORSE MANAGEMENT

1. Does the horse have regular checks by the following professionals and how frequently do they occur?

Professional	Never	Monthly	Bimonthly	Quarterly	Biannually	Annually
Physiotherapist						
Chiropractor						
Dentist						
Vet						
Other therapist						

SHOEING

2. How frequently is the horse trimmed and shod? Every:

2 3 4 5 6 7 8 9 10 weeks

3. What type of shoes does the horse wear and what material are they made out of? (Please select shoe type and appropriate options)

	Shoe type				Material					**Extras**		
	None	Standard	Bar shoe	Other	Aluminium	Steel	Plastic	Pads Yes/ No	Packing Yes/ No	Rolled toes	graduated	Lat extensions
Front feet												
Hind feet												

4. Please select from the image below which stud type (if any) you would use in different ground conditions for the different phases of competition.

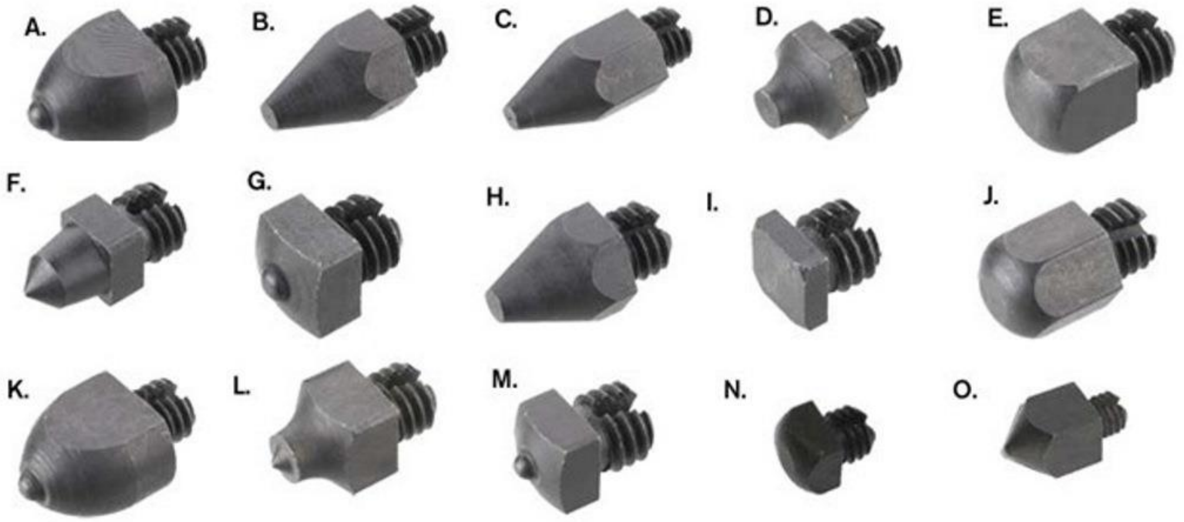

Surface	Cross-country – front feet	Cross-country – hind feet	Showjumping - front feet	Showjumping - hind feet
"Good" grass going				
Firm ground				
Slippery				
Boggy				
Firm ground				
Artificial sand surface				

5. Please select from the image below which stud configuration you would use in different ground conditions for the different phases of competition. (Please leave blank if studs are not used for a specific type of surface)

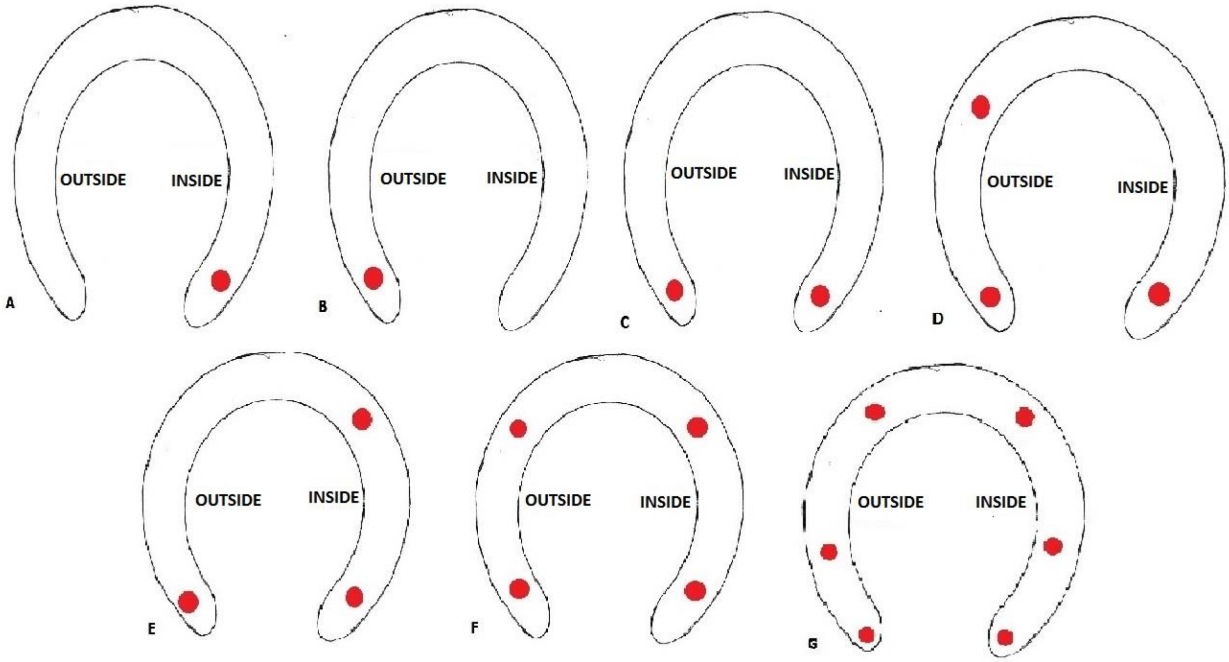

Surface	Cross-country – front feet	Cross-country – hind feet	Showjumping - front feet	Showjumping - hind feet
"Good" grass going				
Firm ground				
Slippery				
Boggy				
Firm ground				
Artificial sand surface				

STABLING

6. During the competition season, approximately how many hours does the horse spend turned out:

None

Less than 5

6 – 12

More than 12, but not full time

More than 12, including full time

TRAVEL

7. During the competition season, what was the average length (one way) travelled to a BE competition? ____________________________

TACK

8. What types of tack do you use for the following types of exercise?

Exercise	Bridle	Noseband	Breastplates	Martingale	Saddle	Comments
Flat work						
Showjumping (training)						
Cross-country (training)						
Fast work (inc. interval training)						
Dressage (Competition)						
Showjumping (Competition)						
Cross-country (Competition)						

9. When was the saddle fit last checked (please select most applicable):

0-3 months 3-6 months 6-9 months 9-12months More than 12months

a) Who was it checked by?

Qualified saddle fitter Master saddler Physiotherapist Vet Trainer

Other (please specify) _________

BOOTS

11. What type of limb protection do you use?

Boot type	Forelimbs		Hindlimbs	
	Cross-country	Showjumping	Cross-country	Showjumping
Over-reach boots				
Brushing boots				
Cross-country boots				
Tendon boots				
Fetlock boots				
Bandages				
Other				

12. Have you had problems with using boots? Yes No

a) If Yes, what were the problems? (select all that apply)
   Rubbing
   Slipping
   Pressing on tendons
   Restricting leg movement
   Getting too heavy if wet
   Damage due to overtightening
   Boots too long for leg
   Boots too short for leg
   Other (please specify) _______________

ONGOING VETERINARY SUPPORT/TREATMENT

13. Were the horse's joints medicated during the season? Yes No

a) If Yes, please answer which area(s) were medicated and how many times?

**Joint**	Number of times medicated
Hock	
Coffin joints (front)	
Coffin joints (hind)	
Fetlock joint (front)	
Fetlock joint (hind)	
Stifle	
Carpus (knee)	
Pelvis/Sacroiliac	
Back	
Pastern joints (front)	
Pastern joints (hind)	
Shoulder	
Elbow	
Tendon sheath	
Other	

SECTION 5: TRAINING AND WORK RELATED INFORMATION

1. How long does the horse spend being exercised each week?

Exercise	Sessions per week	Minutes per session (if known)				
		0-15 min	16-30 min	31-45 min	46-60 min	more than 60 min
Flat work						
Showjumping						
Cross-country schooling						
Fast work (inc. interval training)						
Hacking						
Horse walker						
Land/Dry Treadmill						
Water Treadmill						
Lungeing/Long-reining						
Pole work (in-hand)						
Pole work (ridden)						

2. Who is the primary rider/handler for the following training/exercise?

Exercise	Competitor	Work rider	Groom	Other
Flat work				
Showjumping				
Cross-country				
Fast work (inc. interval training)				

WARM UP AND WARM/COOL DOWN

3. How long (in minutes) is a general warm-up session before training and warm/cool down session after training?

	Flatwork	Showjumping	Cross country schooling	Fast work
Warm-up	a) 0-5 min<br>b) 6-10 min<br>c) 11-15 min<br>d) 16-20 min<br>e) over 20 min	a) 0-5 min<br>b) 6-10 min<br>c) 11-15 min<br>d) over 15 min	a) 0-5 min<br>b) 6-10 min<br>c) 11-15 min<br>d) over 15 min	a) 0-5 min<br>b) 6-10 min<br>c) 11-15 min<br>d) over 15 min
Warm/ cool down	a) 0-5 min<br>b) 6-10 min<br>c) 11-15 min<br>d) 16-20 min<br>e) over 20 min	a) 0-5 min<br>b) 6-10 min<br>c) 11-15 min<br>d) over 15 min	a) 0-5 min<br>b) 6-10 min<br>c) 11-15 min<br>d) over 15 min	a) 0-5 min<br>b) 6-10 min<br>c) 11-15 min<br>d) over 15 min

SECTION 6: TRAINING SURFACES

1. Approximately, how much time (in minutes per week) is spent doing roadwork at the start, middle and end of the season?

	Duration at walk (in minutes)	Duration at trot (in minutes)
Start of the season	a) 0<br>b) 1-15 min<br>c) 16-30 min<br>d) 31-45 min<br>e) 46-60 min<br>f) 1 - 2 h<br>g) more than 2 h	a) 0<br>b) 1-5 min<br>c) 6-15 min<br>d) 16-30 min<br>e) more than 30 min
Middle of the season	a) 0<br>b) 1-15 min<br>c) 16-30 min<br>d) 31-45 min<br>e) 46-60 min<br>f) 1 - 2 h<br>g) more than 2 h	a) 0<br>b) 1-5 min<br>c) 6-15 min<br>d) 16-30 min<br>e) more than 30 min
End of the season	a) 0<br>b) 1-15 min<br>c) 16-30 min<br>d) 31-45 min<br>e) 46-60 min<br>f) 1 - 2 h<br>g) more than 2 h	a) 0<br>b) 1-5 min<br>c) 6-15 min<br>d) 16-30 min<br>e) more than 30 min

2. Please indicate how frequently the horse trains on each of the following surfaces:

Grass ______ times per week
Wax/gel coated ______times per week
Sand and Fibre ______ times per week
Sand and Rubber ______ times per week
Sand only ______times per week
Fibre only ______ times per week
Woodchip ______times per week
Other (Please specify) ____________________

3. What surface type do you most frequently perform the following exercises on? Please select only one per exercise.

Exercise	Artificial surface	Grass	Variable	Other (please specify)
Flat work/Dressage				
Showjumping				
Cross-country				
Fast work (inc. interval training)				

SECTION 7: COMPETITION INFORMATION

FOR COMPETITIONS IN THE LAST 12 MONTHS.

1. What was the horse's highest ranked finish?
2. Does this horse normally finish in the top, middle or bottom third?
3. Do you regularly get time penalties on the cross-country phase? Yes No

3a. If YES, is it: a) Faster than time b) Slower than time c) Both

4. Has this horse had any injuries or health problems (eg tying up, overheating, dehydrated, nose bleed, collapse) AT a specific event?
(Please complete relevant boxes in the table)

		INJURIES	OTHER HEALTH PROBLEMS
Injury/ health problem details			
Event details (e.g. CHILHAM CASTLE April)			
Phase of the competition	Dressage		
	Showjumping		
	Cross-country		
Ground conditions	Hilly		
	Deep		
	Hard ground		
	Rough ground		
	Variable		
	Other		
Weather conditions	Sunny		
	Low sun		
	Overcast		
	Windy		
	Light rain		
	Heavy Rain		
Travel time to the event			

4a. Was this injury/health problem associated with a fall? Yes No

5. Has this horse had any injuries or health problems (eg tying up, overheating, dehydrated, nose bleed, collapse) AFTER a specific event?

(Please complete relevant boxes in the table)

		INJURIES	OTHER HEALTH PROBLEMS
Injury/ health problem details			
Event details (e.g. CHILHAM CASTLE April)			
Phase of the competition	Dressage		
	Showjumping		
	Cross-country		
Ground conditions	Hilly		
	Deep		
	Hard ground		
	Rough ground		
	Variable		
	Other		
Weather conditions	Sunny		
	Low sun		
	Overcast		
	Windy		
	Light rain		
	Heavy Rain		
Travel time to the event			

MOST RECENT FALLS IN COMPETITION IN LAST 12 MONTHS.

6. Select all that apply to most recent falls involving this horse:

		RIDER FALL	COMBINATION FALL
Total number of falls			
Event of most recent fall (e.g. CHILHAM CASTLE April)			
Phase of the competition	Dressage		
	Showjumping		
	Cross-country		
Ground conditions (select all applicable)	Hilly		
	Deep		
	Hard ground		
	Muddy		
	Ridge and furrow		
	Rough ground		
	Slippery		
	Variable		
	Other		
Weather conditions (select all applicable)	Sunny		
	Low sun		
	Overcast		
	Windy		
	Light rain		
	Heavy Rain		
Travel time to the event			

7. Did the fall occur at a fence? Yes No, If YES:

7a. What fence type did the fall occur?

Arrowhead	Bank	Brush fence	Coffin
Corner	Ditch	Drop fence	Log fence
Normandy bank	Rolltop	Shark's tooth	Skinny
Stone wall	Sunken road	Table	Trakehner
Water			

7b. Was the fence a part of a combination? Yes No

7c. Was it a rotational fall? Yes No

7d. What happened? (select all applicable)

problem on approach problem at take-off problem on landing

hit with front legs | hit with hind legs |
--- | --- | ---

frangible pins broke | frangible pins did NOT break | other

7e. What were the ground conditions on the approach? (Please select all that are applicable)

Dry/firm | Slippery | Deep | Boggy

Standing water | Uphill | Downhill | Flat

7f. What were the ground conditions on the landing? Please select all that are applicable

Dry/firm | Slippery | Deep | Boggy

Standing water | Uphill | Downhill | Flat

Not applicable

7g. Were there any injuries?
Horse | Rider | Not applicable

Do you have any further comments on things you think might increase risk factors for injury and falls in Eventing horses?

To be entered in the prize draw please leave your email below:

THANK YOU FOR TAKING THE TIME TO COMPLETE THIS QUESTIONNAIRE
YOU HAVE PLAYED A VALUABLE PART IN THE FUTURE OF BRITISH EVENTING

WorldHorseWelfare BRITISH EVENTING Providing the ultimate equestrian challenge AHT Animal Health Trust BRITISH EVENTING Charitable FOUNDATION BRITISH EQUESTRIAN FEDERATION uk sport LOTTERY FUNDED
